# Post-operative acute kidney injury in non-suprainguinal vascular surgery patients with a pre-operative history of hypertension

**DOI:** 10.17179/excli2015-103

**Published:** 2015-03-02

**Authors:** Yoshan Moodley, Bruce M. Biccard

**Affiliations:** 1 Peri-operative Research Group, Department of Anaesthetics, Nelson R. Mandela School of Medicine, University of Kwazulu-Natal, Private Bag X7, Congella 4013, South Africa; 2 Inkosi Albert Luthuli Central Hospital, Durban, South Africa

**Keywords:** Acute kidney injury, vascular surgery, hypertensive

## Abstract

Hypertension is an independent predictor of acute kidney injury (AKI) in non-cardiac surgery patients. There are a few published studies which report AKI following non-suprainguinal vascular procedures, but these studies have not investigated predictors of AKI, including anti-hypertensive medications and other comorbidities, in the hypertensive population alone. We sought to identify independent predictors of post-operative AKI in non-suprainguinal vascular surgery patients with a pre-operative history of hypertension. We performed univariate (chi-squared, or Fisher's exact testing) and multivariate (binary logistic regression) statistical analysis of prospectively collected data from 243 adult hypertensive patients who underwent non-suprainguinal vascular surgery (lower limb amputation or peripheral artery bypass surgery) at a tertiary hospital between 2008 and 2011 in an attempt to identify possible associations between comorbidity, acute pre-operative antihypertensive medication administration, and post-operative AKI (a post-operative increase in serum creatinine of ≥ 25 % above the pre-operative measurement) in these patients. The incidence of post-operative AKI in this study was 5.3 % (95 % Confidence Interval: 3.2-8.9 %). Acute pre-operative β-blocker administration was independently associated with post-operative AKI in non-suprainguinal vascular surgery patients with a pre-operative history of hypertension (Odds Ratio: 3.24; 95 % Confidence Interval: 1.03-10.25). The acute pre-operative administration of β-blockers should be carefully considered in non-suprainguinal vascular surgery patients with a pre-operative history of hypertension, in lieu of an increased risk of potentially poor post-operative renal outcomes.

## Introduction

Acute kidney injury (AKI) is a significant cause of in-hospital morbidity and mortality in hospitalized patients (Borthwick and Ferguson, 2010[4]; Chertow et al., 2005[5]). The prevalence of AKI following major non-cardiac surgery varies between 1 % (Kheterpal et al., 2009[10]) and 57 % (Macedo et al., 2008[12]). Hypertension has been identified as an independent predictor of AKI in noncardiac surgery patients (Kheterpal et al., 2009[10]). Amongst vascular surgery patients, most studies describe peri-operative renal outcomes following suprainguinal procedures, namely abdominal aortic aneurysm repair (Thakar, 2013[15]), where an excessively high burden of post-operative renal injury is often reported. Whilst there are studies which report post-operative AKI following non-suprainguinal procedures, such as lower limb amputation and peripheral artery bypass surgery (Adalbert et al., 2013[2]; Arora et al., 2013[3]), these studies have failed to investigate predictors of poor post-operative renal outcomes, including anti-hypertensive medications and other comorbidities, in the hypertensive population alone. We sought to identify independent predictors of post-operative AKI in non-suprainguinal vascular surgery patients with a pre-operative history of hypertension.

## Materials and Methods

This study was a sub-analysis of data from an ethically approved (University of Kwazulu-Natal Biomedical Research Ethics Committee approval reference: BF068/07, BCA117/010) prospective database of adult patients who underwent elective vascular surgery at a tertiary hospital located in Durban, South Africa between 2008 and 2011 (Moodley et al., 2013[14]). We considered a patient to be hypertensive if the patient was diagnosed as having hypertension by a physician, or if the patient was taking any antihypertensive medications (Angiotensin converting enzyme inhibitors - ACEI, β-blockers - βB, or calcium channel blockers - CCB). A total of 243 patients were included in our final analysis, following the exclusion of

**Figure 1:**
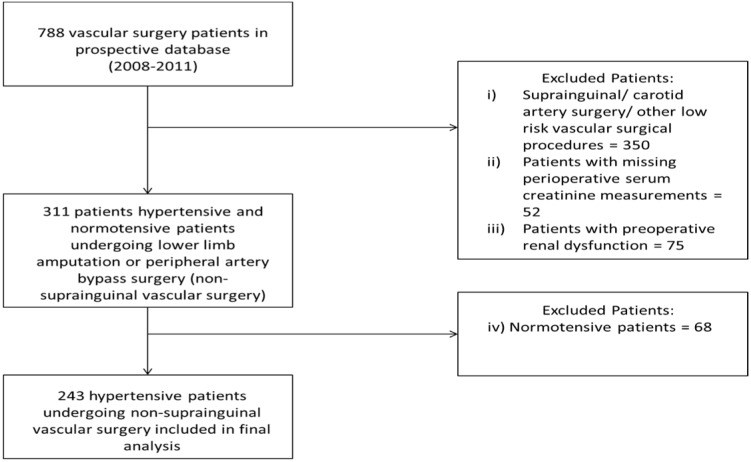
Derivation of the final study cohort

1. patients who did not undergo non-suprainguinal vascular surgery (lower limb amputation or peripheral artery bypass surgery),
2. patients with missing peri-operative serum creatinine measurements,
3. patients with pre-operative renal dysfunction, as defined in the study by Abelha and colleagues (Abelha et al., 2009[1]) as a pre-operative serum creatinine measurement of ≥ 141 µmol/L in men or ≥ 124 µmol/L in women, and
4. patients without a history of hypertension (Figure 1(Fig. 1)).

Data elements collected from patient medical records included demographic information (age and gender), comorbid conditions (history of diabetes, ischaemic heart disease, congestive heart failure, stroke), acute pre-operative anti-hypertensive medication use (ACEI, βB, CCB), and peri-operative laboratory test results (pre- and post-operative serum creatinine measurements). The definitions of comorbid conditions used in this study were adopted from the study of Lee and colleagues (Lee et al., 1999[11]). In this study we defined post-operative AKI as an increase in post-operative serum creatinine ≥ 25 % above the pre-operative measurement. We had chosen this threshold rather than the current Kidney Disease Improving Global Outcomes group defined serum creatinine increase threshold (Kellum and Lameire, 2013[9]) as a large hospital administrative database study found a 25 % increase in serum creatinine from the baseline measurement was associated with a 2-fold increased risk of mortality in hospitalized patients (Chertow et al., 2005[5]). Furthermore, a published meta-analysis found a 3- to 7-fold increase in risk for mortality in hospitalized (surgical and non-surgical) patients with serum creatinine increases of > 25 % from baseline (Coca et al., 2007[6]). Lastly, our definition of AKI is in keeping with the definition used in a meta-analysis of renal outcomes in patients undergoing major surgery (Ho and Morgan, 2009[8]). Use of the less liberal Kidney Disease Improving Global Outcomes group definition (serum creatinine increase of ≥ 26.5 μmol/l from the baseline/pre-operative measurement, or an increase in post-operative serum creatinine to ≥ 1.5 times the baseline/pre-operative measurement) would have therefore excluded patients from the analysis with a prognostically important serum creatinine measurement in the post-operative period. 

Univariate statistical methods used to analyse data included chi-squared and Fisher's Exact testing, where appropriate. For the multivariate statistical analysis, a binary logistic regression model was used to identify independent predictors of post-operative AKI in non-suprainguinal vascular surgery patients with a pre-operative history of hypertension. The clinical variables entered into the logistic regression equation were purposefully selected, with variables attaining a significance level of p <0.1 at the univariate analysis level being included in the regression analysis. Results for the univariate data analysis are presented as frequencies and percentages, whilst results for the binary logistic regression analyses are presented as odds ratios (OR) with 95 % confidence intervals (95 % CI). Any p-value < 0.05 was considered to be a statistically significant result. All statistical analyses were performed using the Statistical Package for the Social Sciences (SPSS) version 21 (SPSS Inc., Chicago, IL, USA).

## Results

The process by which patients were selected for inclusion in this study is shown in Figure 1(Fig. 1). Thirteen of the 243 (5.3 %; 95 % CI: 3.2-8.9 %) patients included in our study developed post-operative AKI. The baseline characteristics of patients included in our final analysis are shown in Table 1(Tab. 1). A large proportion of patients who underwent surgery in the entire cohort were elderly. In addition, almost two-thirds of the entire cohort was male. Diabetes and ischaemic heart disease were the most prevalent comorbid conditions in the entire cohort. With regard to anti-hypertensive medication use, it was common for ACEI or βB, and to a lesser extent CCBs, to have been acutely administered prior to a patient's surgery.

When stratified by post-operative AKI status, no statistically significant univariate associations were observed between patient demographic factors, comorbidities and post-operative AKI (Table 1(Tab. 1)). The point estimates for acute pre-operative ACEI and CCB administration showed a trend toward post-operative renal protection in the presence of these anti-hypertensive medications. A statistical trend was also observed for the association between acute pre-operative βB administration and post-operative AKI (p=0.067, Table 1(Tab. 1)). This statistical trend satisfied the inclusion criterion for the entry of a clinical variable into the logistic regression analysis in our study (p<0.1 at the univariate analysis level). When entered into a logistic regression equation, acute pre-operative βB administration was found to be independently associated with post-operative AKI in non-suprainguinal vascular surgery patients who had a pre-operative history of hypertension (OR: 3.24; 95 % CI: 1.03-10.25).

## Discussion

Five of every 100 hypertensive patients undergoing non-suprainguinal vascular surgery in this study suffered post-operative AKI. The incidence of post-operative AKI in our study was lower than that reported for two other studies of non-suprainguinal vascular surgery, which reported the incidence of post-operative AKI to be 12 % and 12.7 % (Adalbert et al., 2013[2]; Arora et al., 2013[3]). Pre-operative renal dysfunction is an important determinant of post-operative renal outcomes. Although a lower incidence of post-operative AKI in the combined hypertensive-normotensive populations described in the aforementioned studies would be expected, our study population was different in that we had investigated renal outcomes in patients with normal pre-operative renal function, and excluded patients with pre-operative renal dysfunction from our final analysis. However, patients with pre-operative renal dysfunction were included in the two other studies of AKI in non-suprainguinal vascular surgery patients (Adalbert et al., 2013[2]; Arora et al., 2013[3]), thereby explaining the higher incidence of post-operative AKI reported in these studies.

In contrast to other studies of non-cardiac and vascular surgery patients (Adalbert et al., 2013[2]; Arora et al., 2013[3]; Kheterpal et al., 2009[10]), we did not observe associations between any of the patient demographic characteristics or comorbidities and post-operative AKI in our study. It is likely that our study was not adequately powered to determine the impact of these variables on post-operative AKI. Similarly, it appears our study was not adequately powered to investigate the effects of acute pre-operative ACEI and CCB administration on post-operative renal outcomes. It is important to note however, that ACEI and CCB were associated with a trend toward renal protection in the post-operative period, as opposed to acute pre-operative βB use, which was associated with post-operative renal injury.

We found acute pre-operative βB administration to be associated with a 3-fold increased risk of developing post-operative AKI in this study. In another study, we had reported that acute pre-operative βB administration was associated with a higher risk of peri-operative troponin leak and peri-operative major adverse cardiovascular events in vascular surgery patients with a pre-operative history of hypertension (Moodley and Biccard, 2015[13]). In that study we postulated that vascular surgery patients with a pre-operative history of poorly controlled hypertension might be pre-disposed towards peri-operative hypotension. The peri-operative hypotension might have been further exacerbated by acute pre-operative βB administration, resulting in a higher incidence of peri-operative cardiac morbidity and mortality in patients who received acute pre-operative βB versus patients who did not receive acute pre-operative βB in that study (Moodley and Biccard, 2015[13]). Similarly, it is therefore possible that acute pre-operative βB administration exacerbates global peri-operative hypotension in patients with poorly controlled pre-operative hypertension, resulting in ischaemic injury of the kidney. The potentially aggravating effects of acute pre-operative βB administration on global peri-operative hypotension observed in this study are further supported by the findings of the POISE study (Devereaux et al., 2008[7]), wherein acutely administered pre-operative metoprolol was found to be independently associated with a higher incidence of peri-operative hypotension and peri-operative stroke following non-cardiac surgery. The findings of our research have potential implications for peri-operative medication management in vascular surgery patients, particularly those with a pre-operative history of hypertension. Our study was not without limitations. It is likely that our study was underpowered to investigate the impact of patient demographic variables, other comorbidities, and the acute pre-operative use of ACEI and CCB on the incidence of post-operative AKI. A larger patient cohort would be required to adequately investigate this. 

In conclusion, acute pre-operative β-blockade was associated with a higher risk of post-operative AKI in non-suprainguinal vascular surgery patients with a pre-operative history of hypertension. Although this research requires validation in a larger cohort, our findings suggest that the acute pre-operative administration of βB should be carefully considered in non-suprainguinal vascular surgery patients who have a pre-operative history of hypertension.

## Acknowledgements

This study was funded by a South African Medical Research Council self-initiated research grant awarded to Bruce M. Biccard. This work forms a component of the doctoral studies of Yoshan Moodley, who is the recipient of a doctoral scholarship awarded by the South African National Research Foundation (NRF). Yoshan Moodley was also supported by the Columbia University-Southern African Fogarty AIDS International Training and Research Program (AITRP), Implementation Science Scholarship Program funded by PEPFAR through the Fogarty International Center, National Institutes of Health (grant # D43TW000231).

## Conflict of interest

The authors declare that they have no conflict of interest.

## Figures and Tables

**Table 1:**
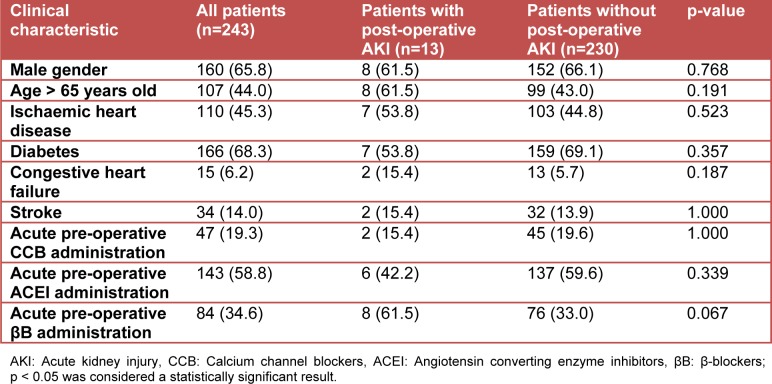
Clinical characteristics of patients with and without post-operative AKI expressed as a frequency (percentage)
